# Revolutionizing Intracranial Hemorrhage Diagnosis: A Retrospective Analytical Study of Viz.ai ICH for Enhanced Diagnostic Accuracy

**DOI:** 10.7759/cureus.66449

**Published:** 2024-08-08

**Authors:** Mona P Roshan, Seema A Al-Shaikhli, Italo Linfante, Thompson T Antony, Jamie E Clarke, Raihan Noman, Chrisnel Lamy, Sean Britton, Starlie C Belnap, Kevin Abrams, Charif Sidani

**Affiliations:** 1 Radiology, Florida International University, Herbert Wertheim College of Medicine, Miami, USA; 2 Miami Neuroscience Institute, Baptist Health South Florida, Miami, USA; 3 Radiology, University of Miami Miller School of Medicine, Miami, USA; 4 Epidemiology and Biostatistics, Florida International University, Herbert Wertheim College of Medicine, Miami, USA; 5 Radiology, Florida State University, Miami, USA; 6 Radiology, Baptist Health South Florida, Miami, USA

**Keywords:** radiology and imaging, intraparenchymal hemorrhage, artificial intelligence in radiology, intraventricular hemorrhage, subarachnoid hemorrhage, subdural hemorrhage, artificial intelligence, technology, stroke, intracranial hemorrhage (ich)

## Abstract

Introduction

Artificial intelligence (AI) alerts the radiologist to the presence of intracranial hemorrhage (ICH) as fast as 1-2 minutes from scan completion, leading to faster diagnosis and treatment. We wanted to validate a new AI application called Viz.ai ICH to improve the diagnosis of suspected ICH.

Methods

We performed a retrospective analysis of 4,203 consecutive non-contrast brain computed tomography (CT) reports in a single institution between September 1, 2021, and January 31, 2022. The reports were made by neuroradiologists who reviewed each case for the presence of ICH. Reports and identified cases with positive findings for ICH were reviewed. Positive cases were categorized based on subtype, timing, and size/volume. Viz.ai ICH output was reviewed for positive cases. This AI model was validated by assessing its performance with Viz.ai ICH as the index test compared to the neuroradiologists' interpretation as the gold standard.

Results

According to neuroradiologists, 9.2% of non-contrast brain CT reports were positive for ICH. The sensitivity of Viz.ai ICH was 85%, specificity was 98%, positive predictive value was 81%, and negative predictive value was 99%. Subgroup analysis was performed based on intraparenchymal, subarachnoid, subdural, and intraventricular subtypes. Sensitivities were 94%, 79%, 83%, and 44%, respectively. Further stratification revealed sensitivity improves with higher acuity and volume/size across subtypes.

Conclusion

Our analysis indicates that AI can accurately detect ICH's presence, particularly for large-volume/large-size ICH. The paper introduces a novel AI model for detecting ICH. This advancement contributes to the field by revolutionizing ICH detection and improving patient outcomes.

## Introduction

Stroke, recognized as the second-leading cause of death and the third-leading cause of death and disability globally, imposes a significant health burden [1]. A stroke occurs when blood flow to the brain is disrupted, either by a blockage (ischemia) or by a burst blood vessel (hemorrhage), resulting in brain cell damage. Among the various types of strokes, intracranial hemorrhage (ICH) constitutes a critical subset, accounting for 10-20% of all strokes annually [2,3]. Although a small percentage, this acute neurological emergency has a disproportionately high risk of brain injury, long-term disability, and death. ICH's one-month morbidity and mortality is approximately 50% [4]. Additionally, over 75% of ICH patients either die or suffer severe disability [4]. Brain injury from non-traumatic etiologies, such as ruptured cerebral aneurysms or vascular malformations, is also associated with high morbidity or mortality. Increased blood volume and subsequential intracranial pressure can cause brain herniation without immediate diagnosis and treatment, leading to death or permanent tissue damage [5]. Therefore, ICH requires effective, accurate, and prompt diagnosis to be minimized and appropriately treated, along with preventing complications.

ICH can be subclassified into two main categories, intra-axial and extra-axial, which themselves are even further subdivided. The intra-axial class consists of intraventricular and intraparenchymal hemorrhages. The extra-axial class is comprised of subarachnoid, subdural, and epidural hemorrhages [5,6]. Subarachnoid hemorrhages result from the rupture of vessels in the subarachnoid space overlying the brain parenchyma. These vessels most commonly arise from arteriovenous or aneurysm malformation. Epidural hemorrhage is caused by trauma and is found in the epidural space above the dura mater. Subdural hemorrhage is within the subdural space between the arachnoid and pia mater and below the dura mater. Altogether, these various forms of ICH can be life-threatening and require urgent medical attention.

The gold standard and commonly used method to diagnose ICH is non-contrast computed tomography (CT) [7]. It is the diagnostic test of choice for ICH because of its remarkable sensitivity, specificity, and wide availability [8,9]. Magnetic resonance imaging (MRI) has also been utilized in more problematic traumatic brain injury cases, especially since recent studies have shown promising sensitivities [6,8]. Accurate and timely detection of ICH is crucial to making critical treatment decisions and providing prompt life-saving measures [10].

Medicine has grown increasingly interested in machine learning (ML) techniques due to their high accuracy and speed [11]. ML is a form of artificial intelligence (AI) that uses data to learn patterns and rules [4]. AI alerts the radiologist to the presence of hemorrhage within 1-2 minutes from scan completion, leading to faster diagnosis. As an adjunct to physicians throughout medicine, AI enhances diagnostic accuracy and reduces physicians' workload [12]. The application of AI detecting ICH has become increasingly prevalent as imaging utilization continues to rise for various intracranial pathologies [10,12-14]. These include but are not limited to ICH, intracranial aneurysms, brain tumors, and traumatic brain injury.

The purpose of this study is to validate a new AI application called Viz.ai ICH with the intent to enhance and expedite the diagnosis of suspected ICH. Viz.ai ICH is an AI-powered software for detecting and triaging ICH, with advanced AI algorithms that detect and localize ICH in real time. This AI model was trained on all forms of ICH, including epidural, subdural, subarachnoid, and intraparenchymal hemorrhage. The Food and Drug Administration (FDA) currently approves two hemorrhage algorithms: one for subdural hemorrhage (Viz.ai SDH) and another for all hemorrhages (Viz.ai ICH). In contrast to existing studies, this study aimed to determine the sensitivity and specificity of this AI model by analyzing the cases in sum and subcategories of the ICH subtype along with timing, volume, and size. Our hypothesis is that Viz.ai has high sensitivity and specificity, leading to faster and more accurate detection and treatment of ICH.

## Materials and methods

In this study, we retrospectively analyzed the database of a single institution between September 1, 2021, and January 31, 2022, for patients of any age and gender presenting with any symptoms in which a non-contrast brain CT was performed. All scans were performed on General Electric multi-slice CT scanners (Milwaukee, WI) acquired axial at 2.5 mm, with sagittal and coronal reformats obtained. Inclusion criteria included all CT scans of the brain performed at the institution between September 1, 2021, and January 31, 2022, all genders, all age groups, and in-patient/emergency department/out-patient settings. This included non-contrast-enhanced CT brain, non-contrast-enhanced CT brain as part of computed tomography angiography (CTA), and non-contrast-enhanced CT brain as part of CT with contrast. Exclusion criteria included non-diagnostic exams, as determined by radiologists. Baseline patient characteristics were retrieved from the electronic medical record. According to the inclusion and exclusion criteria, 4,203 consecutive non-contrast brain CT reports were included, and zero were excluded.

A team of experienced neuroradiologists made reports after thoroughly reviewing each case for the presence and absence of ICH. Neuroradiologist reviewers were blinded for clinical information and primary reads. Neuroradiologists' reports were reviewed, and cases indicative of ICH were identified. The radiologists' impressions were recorded for each positive case. They later reviewed the Viz.ai reports for their assigned months and noted the cases the Viz.ai model predicted as ICH. For any discordance between the radiologist's report and Viz.ai, any false positive on Viz.ai was reviewed by a neuroradiologist with 10 years of experience. All true positive cases were examined to quantify hemorrhage. A randomized group of ICH-positive cases was categorized based on subtype, timing, and size/volume through imaging review.

The subtypes included intraparenchymal, subarachnoid, subdural, and intraventricular. The timing was subcategorized as either subacute or acute for cases with positive findings for intraparenchymal, subarachnoid, and intraventricular hemorrhages. For subdural hemorrhages, the subcategories for timing included subacute, acute, chronic, and mixed. Volume was also a subcategory: minimal, moderate, or large for intraventricular hemorrhage, minimal, moderate, large, or diffuse for subarachnoid hemorrhage, and >5 cc or <5 cc for intraparenchymal hemorrhage. The largest anterior-posterior (AP), craniocaudal (CC), and transverse (TR) dimensions were measured by the radiologist, and the volume was calculated as (AP×CC×TR)/2. The rationale for volume categories was subjective based on neuroradiologist expertise and discussion for interobserver agreement. The cases with positive findings for subdural hemorrhages were also subcategorized based on size as either >10 mm or <10 mm.

This AI model was validated using descriptive analysis, and its diagnostic performance was assessed. Viz.ai ICH as the index test was compared to the neuroradiologists' interpretation as the gold standard. Using the Stata Statistical Software: Release 17 (2021; StataCorp LLC, College Station, Texas, United States), the sensitivity, specificity, and positive and negative predictive values were found for the total number of cases. In addition, a randomized subset of cases was evaluated for subgroup analysis. The sensitivity and specificity were determined for each subtype of intraparenchymal, subarachnoid, and intraventricular hemorrhages, along with the subcategories of timing, volume, and size, depending on the subtype.

Potential biases were addressed by ensuring a diverse patient population, blinding neuroradiologists to clinical information, and using independent assessments by both radiologists and Viz.ai. Cross-validation techniques confirmed the AI model's performance across different subgroups, ensuring reliable and consistent results. As the primary aim was to evaluate Viz.ai's performance detecting ICH across a diverse patient population, potential confounders and effect modifiers were not identified, so no control measures were implemented.

The study was approved by the Institutional Review Board of Baptist Health South Florida (IRBNet ID 1849788-2 and date of approval 4/4/2022).

## Results

**Figure 1:**
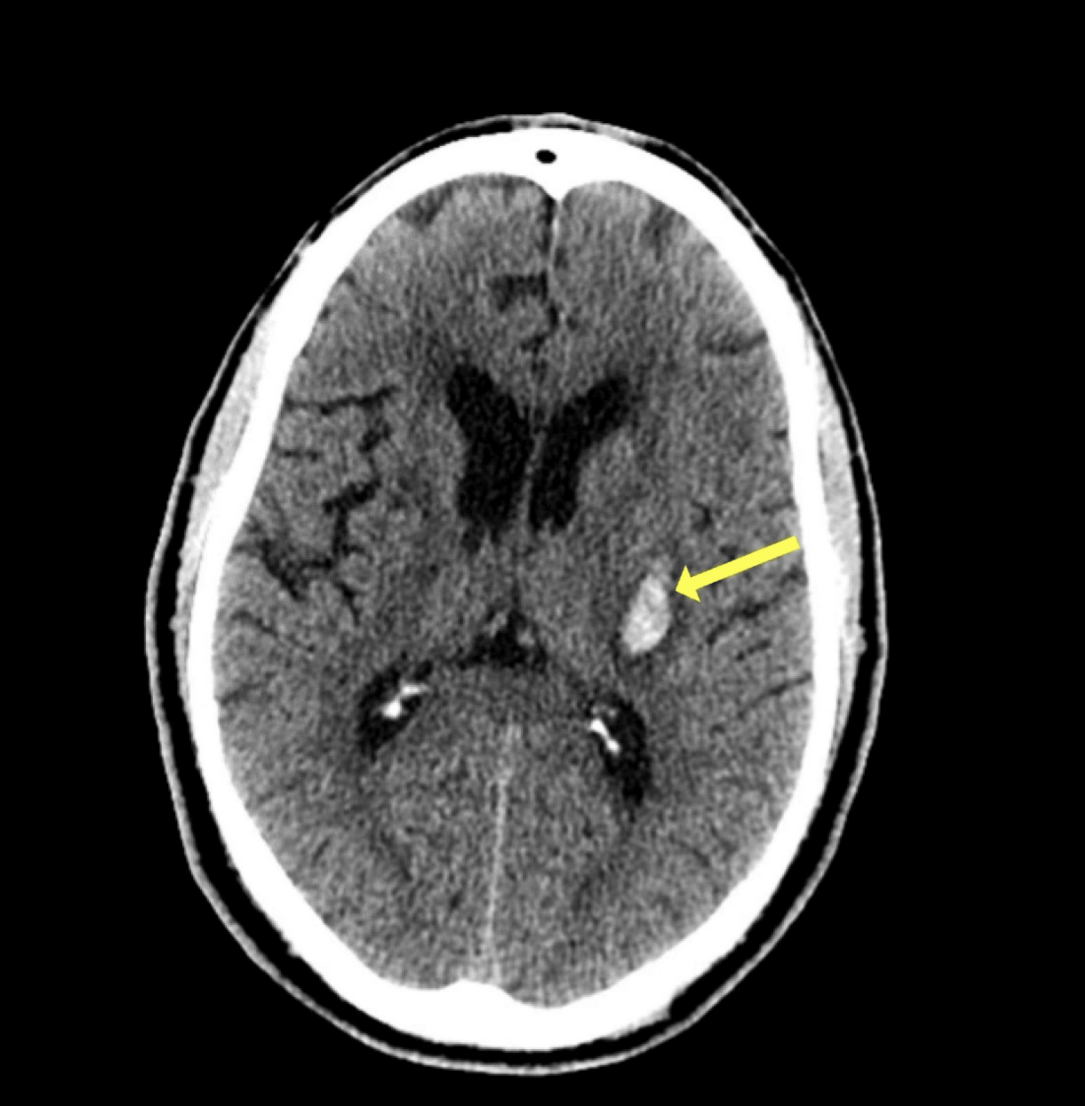
True positive ICH The sensitivity of Viz.ai ICH for acute intraparenchymal hemorrhage was 94%. Acute hemorrhage is in the left basal ganglia (volume <5 cc) ICH: intracranial hemorrhage

The mean age of the included population (4,203 total non-contrast brain CT reports) was 64.6 (SD=20.5), with 55.9% identifying as female, and 9.2% of the retrieved non-contrast brain CTs were positive per the radiology impression. The overall performance of Viz.ai ICH in detecting ICH is summarized in Table 1. The sensitivity and specificity of Viz.ai ICH were 85% and 98%, respectively (Figure 1). The positive predictive value was 81%, while the negative predictive value was 99%.

**Table 1:** Summary of the performance of Viz.ai ICH in the detection of ICH ICH: intracranial hemorrhage; CI: confidence interval; PPV: positive predictive value; NPV: negative predictive value

	Sensitivity	Specificity	PPV	NPV
Overall (95% CI)	85.3% (82.1-88.1%)	98.3% (98.3-98.6%)	81.3% (78-84.4%)	98.7% (98.4-99%)

A randomized group of non-contrast brain CTs were included in intraparenchymal, subarachnoid, subdural, and intraventricular hemorrhagic subgroups. Following subgroup analysis of a randomized subset of cases, sensitivities were recalculated to be 94% for intraparenchymal hemorrhage, 79% for subarachnoid hemorrhage, 83% for subdural hemorrhage, and 44% for intraventricular hemorrhage.

Further stratification improved sensitivity with higher acuity and volume/size across all ICH subtypes. Intraparenchymal, subarachnoid, and intraventricular hemorrhages demonstrated higher sensitivity in acute ICH (98%, 92%, 83%) than in subacute ICH (69%, 40%, 22%). Within the subdural hemorrhage subgroup, subacute ICH (100%) exhibited higher sensitivity than chronic (33%) and mixed ICH (83%). Concerning volume, intraparenchymal ICH greater than 5 cc (99%) showed higher sensitivity than ICH less than 5 cc (84%). For subarachnoid and intraventricular ICH, sensitivity was higher for large (100% for both) and moderate volume (100% for both) compared to minimal volume (74% and 50%, respectively). Subarachnoid ICH of diffuse volume also demonstrated lower sensitivity (80%) than ICH of definitively larger volumes. Regarding size for subdural hemorrhage, Viz.ai ICH exhibited higher sensitivity for ICH larger than 10 mm (90%) than ICH smaller than 10 mm (77%). Table 2 demonstrates the results of this subgroup analysis.

**Table 2:** Summary of subgroup analysis on the sensitivity of Viz.ai ICH for different ICH subtypes in detecting ICH ICH: intracranial hemorrhage

ICH subgroup	Sensitivity (N)
Intraparenchymal	94% (136)
Timing	
Acute	98% (118)
Subacute	69% (16)
Volume	
≤5 cc	84% (45)
>5 cc	99% (78)
Subarachnoid	79% (47)
Timing	
Acute	92% (36)
Subacute	40% (10)
Volume	
Minimal	74% (31)
Moderate	100% (8)
Large	100% (2)
Diffuse	80% (5)
Subdural	83% (108)
Timing	
Acute	88% (59)
Subacute	100% (4)
Chronic	33% (9)
Mixed	83% (40)
Size	
≤10 mm	77% (57)
>10 mm	90% (48)
Intraventricular	44% (16)
Timing	
Acute	83% (6)
Subacute	22% (9)
Volume	
Minimal	50% (10)
Moderate	100% (1)
Large	100% (1)

**Figure 2:**
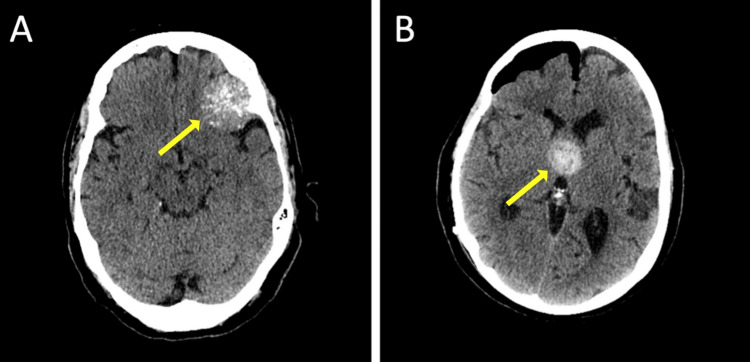
The specificity of Viz.ai ICH was 98%, accounting for a 2% false positive rate Meningioma (A) was the most common false positive finding. An additional example includes thrombosed aneurysm (B) ICH: intracranial hemorrhage

We reviewed the images with false positive detections. Meningioma (14%) was a common false positive finding (Figure 2A). Other false positive findings included thrombosed aneurysm (Figure 2B), calcifications, hyperdense mass, venous congestion from dural arteriovenous fistulas (DAVF), anoxic brain injury, and dense middle cerebral artery (MCA).

## Discussion

In this study, we validated the ability of the Viz.ai ICH AI model to detect the presence of ICH accurately. The algorithm provided high discriminatory accuracy in distinguishing between the presence and absence of ICH on non-contrast brain CT scans. This study involved a large, diverse imaging dataset of 4,203 non-contrast brain CT scans acquired consecutively over five months at a large hospital in South Florida with an active Neuroscience Institute.

Compared to the gold standard of the radiologist impression, the diagnostic performance of Viz.ai ICH was promising, particularly for large-volume/large-size ICH. According to the total data analysis, the negative predictive value of ICH detection was 99%, which meant that most non-contrast brain CTs detected as negative for ICH by Viz.ai were truly negative according to the radiologist's impression. Additionally, the overall positive predictive value was 81%, which meant that most non-contrast brain CTs detected as positive for ICH by Viz.ai were truly positive. The overall specificity was 98%, correlating to a 2% false positive rate. This supports the notion that Viz.ai enhances radiologists' workflow by correctly detecting non-contrast brain CTs as negative for ICH.

While Viz.ai's sensitivity was 85%, subgroup analysis revealed that this number increased to 94% for intraparenchymal hemorrhages. Within each subgroup, the sensitivity of Viz.ai rose for higher acuity and larger-volume/larger-size cases. Thus, we validated Viz.ai's ability to detect the more acute, significant cases of ICH. 

Since missed ICH is devastating and often fatal, a tool that automatically detects this pathology and thus augments the quality of patient care is desirable. Along with improving interpretation accuracy, the Viz.ai application can prioritize workflow, signaling the neuroradiologist to read cases detected as positive by Viz.ai first. This would facilitate timely expert review of significant cases, quick treatment time, and positive patient results. Specifically, it would expedite the speed by which patients are triaged for medical versus surgical management of ICH [15]. 

In facilitating the expedited review process with the Viz.ai application, its automated detection and prioritization features play a crucial role. The algorithm promptly identifies positive ICH cases and sends an alert to the radiologist via a web-based or phone-based app within two minutes from study completion. When integrated with Picture Archiving and Communication Systems (PACS), the software aims to send positive cases to the top of the list, optimizing workflow by streamlining the neuroradiologist's review process. This prioritization ensures that cases with potential clinical significance, such as ICH, are swiftly brought to the medical team's attention. In this way, the Viz.ai application not only enhances the accuracy of interpretation but also contributes to the efficiency of the diagnostic workflow, ultimately benefiting patient care.

Meningiomas were the most common cause of false positive findings upon imaging review of the Viz.ai application. It should also be noted that while false positives occurred, many were for significant findings such as large mass, thrombosed vessel, or aneurysm, for which expedited review would still benefit patient care.

The novel AI technology is an essential medical tool that is transformative in diagnostic capabilities. In addition to validating the Viz.ai ICH AI model, it is crucial to contextualize its performance within the broader landscape of AI applications for ICH detection. Over the past decade, as numerous studies have meticulously scrutinized the diagnostic accuracy of AI models across various medical conditions, ICH detection has witnessed only a limited number of validations. Notably, Chang et al. conducted a rigorous assessment of the convolutional neural network in 2018, showcasing its custom architecture for accurate detection and quantification of various hemorrhage types on non-contrast CT scans, achieving high performance with accuracy, sensitivity, and specificity exceeding 95% [16].

Furthermore, another significant advancement was marked by the evaluation of RAPID ICH, an automated hybrid 2D-3D convolutional neural network application designed to rapidly detect ICHs on head non-contrast CT scans [17]. The findings from this study highlight the exceptional accuracy of RAPID ICH in identifying and volumetrically quantifying intraparenchymal and intraventricular hemorrhages. This showcases the potential for AI to expedite the diagnostic process in busy hospital settings.

Additionally, a study by Ye et al. introduces a novel application of artificial recurrent neural networks for continuous intracranial pressure prediction in traumatic brain injury patients [18]. The proposed model achieves high accuracy (94.62%) and sensitivity (74.91%), addressing the crucial need for real-time early intracranial pressure detection.

Moreover, a study aimed to determine the institutional diagnostic accuracy of the AI decision support system Aidoc in diagnosing ICH on non-contrast head CTs. The retrospective study, encompassing 3,605 consecutive emergent adult scans, found concordance in interpretations between neuroradiologists and Aidoc in 96.9% of cases [19]. Despite an overall sensitivity of 92.3% and specificity of 97.7%, unexpectedly lower positive predictive values raised concerns about the tool's generalizability. Factors such as prior neurosurgery, type of ICH, and the number of ICHs were associated with decreased model performance. This underscores the importance of standardized study design for reliable site-to-site comparisons of emerging deep learning technologies, as discrepancies in diagnostic accuracy were identified within the institutional context.

The Caire ICH system represents another significant advancement in AI for cerebrovascular disease diagnosis [20]. Analyzing 402 head non-contrast CT scans, the system demonstrated an impressive accuracy of 98.05%, sensitivity of 97.52%, and perfect specificity of 100% in detecting ICH and its subtypes. This suggests that AI tools hold potential as reliable point-of-care tools, minimizing clinical errors in ICH diagnosis and improving patient outcomes and radiologists' workflows.

VeriScout™, an AI-based CT hemorrhage detection and triage tool, also exhibited promising real-world performance in diagnosing ICH [21]. Analyzing 527 non-contrast head CT scans, VeriScout™ demonstrated a sensitivity of 92% and specificity of 96%, surpassing general radiologists in sensitivity. Notably, it effectively detected subarachnoid hemorrhage, a critical condition often missed in emergencies. The tool maintained robust performance in scans with artifacts or postoperative changes. Integrated into the radiology workflow, VeriScout™ flagged detected cases in the hospital radiology information system (RIS) and provided annotated preview images in the PACS within 10 minutes. This study highlights the potential of AI-based worklist prioritization to enhance ICH diagnosis without burdening radiologists or clinicians.

Another study validated a commercially available deep learning-based tool called CINA v1.0 device for detecting ICH [22]. The tool demonstrated high accuracy, sensitivity, and specificity for both ICH (95.6%, 91.4%, 97.5%) and large-vessel occlusion (LVO) (98.1%, 98.1%, 98.2%). Despite some limitations, the study concluded that deep learning tools could effectively assist radiologists in detecting emergent findings in various practice settings.

**Figure 3:**
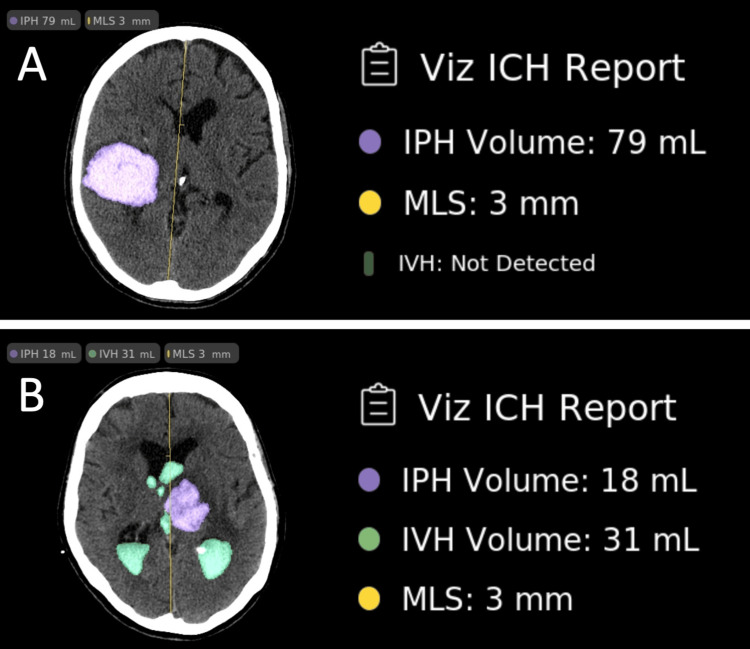
The interface of the latest version of Viz.ai (A) IPH with a volume of 79 mL and an MLS of 3 mm. (B) IPH and IVH with volumes of 18 mL and 31 mL, respectively, along with an MLS of 3 mm IPH: intraparenchymal hemorrhage; MLS: midline shift; IVH: intraventricular hemorrhage

The software has undergone several updates since our initial review, showcasing significant enhancements in its support for patient care. One notable improvement is the introduction of midline shift (MLS) detection. This feature adds the ability to assess whether or not an MLS is present and, if so, to quantify the extent of the shift. Additionally, the software now offers volumetric volume analysis, providing a more comprehensive understanding of the data. In its more recent versions, outside the scope of FDA approval, the software has further expanded its capabilities to include the detection of subdural hematoma. This continuous evolution reflects the commitment to staying at the forefront of medical imaging technology. As part of our ongoing efforts, we are actively engaged in the validation analysis of these new features to ensure their accuracy and reliability in clinical settings. Figure 3 visually represents the latest features incorporated into the app.

Future directions in research for ICH detection using AI are likely to focus on refining and expanding the capabilities of existing models [23-25]. Researchers may aim to enhance sensitivity for smaller pathologies and rare conditions while addressing specific challenges, such as reducing false positives, particularly for conditions like meningiomas that mimic stroke. Integrating AI with advanced imaging modalities and wearable devices could be explored to provide a more comprehensive and continuous monitoring approach [26]. Collaborative efforts between AI developers, healthcare institutions, and regulatory bodies will be crucial to establish standardized study designs and ensure the reliability and generalizability of AI models across diverse settings.

Moreover, future research may delve into the role of AI not only in detection but also in treatment planning and monitoring, contributing to a more holistic patient care approach [27]. The expedited review process facilitated by AI applications, like Viz.ai, could be further optimized to prioritize cases efficiently, ensuring timely expert review of significant findings. Evaluating the impact of AI on the triage of patients for medical versus surgical management of ICH and its implications for positive patient outcomes will likely be an area of interest [28,29].

Additionally, research efforts may explore ethical considerations, user acceptance, and the potential socioeconomic impact of widespread AI integration in neuroradiology [30]. Ongoing optimization, validation, and adherence to regulatory standards will remain crucial to building trust among healthcare professionals and patients, ensuring responsible and effective integration of AI tools into routine clinical practice [31]. The dynamic landscape of AI research in neuroradiology suggests a continuous commitment to refining algorithms and exploring innovative applications to advance diagnostic capabilities further and ultimately improve patient outcomes.

AI is a valuable tool in enhancing medical practice across various specialties, including radiology. By leveraging AI to verify critical findings and complexities, radiologists can augment their diagnostic capabilities and streamline patient care pathways. The systematic organization of lesions and the establishment of priorities facilitate a more efficient interpretation of imaging studies, ultimately optimizing workflow efficiency for radiologists and clinicians. Furthermore, expanding AI's database holds promise for revolutionizing the future of medicine as a whole by enabling more accurate diagnoses, personalized treatment plans, and improved patient outcomes.

Limitations

Despite our study's promising results, several limitations must be addressed. Firstly, the retrospective design of the study constrains its scope. Furthermore, variations in interpretation may have arisen as a different neuroradiologist read each case. Additionally, using imaging data from various CT scanners introduces the possibility of discrepancies in Viz.ai's performance. Moreover, the study's applicability is limited as data was collected solely from a single institution, and findings may not be generalized to institutions with different imaging scanners and protocols.

## Conclusions

Our study indicates that Viz.ai ICH can detect ICH with promising performance. The sensitivity and specificity for this indication are 85% and 98%, respectively. At the time of the case review, Viz.ai was only approved for acute intraparenchymal hemorrhage detection. Compared to the gold standard of the neuroradiologist's impression, the AI model can accurately detect ICH on non-contrast brain CT, particularly for ICH of larger volume/size.

Outside the FDA-approved applications, the software's performance was relatively sensitive. Since our analysis, the algorithm has been updated multiple times, and the software is now FDA-approved for subdural hemorrhage detection. We will reanalyze our data with Viz.ai's updated software. With improvements in the AI algorithm, radiologists can detect various types of ICH more accurately, efficiently, and effectively to minimize lives lost due to stroke.
